# Right Atrium Large Masses Due to Endocarditis Following Permcath Insertion After Covid-19-Induced Renal Failure

**DOI:** 10.30699/ijp.2025.2068882.3506

**Published:** 2025-11-11

**Authors:** Seyed Mohsen Mirhosseini, Hossein Yarmohammadi, Arash Anisian, Fatemeh Ravand, Mahdi Rezaei, Masood Soltanipu

**Affiliations:** 1 *Cardiovascular Research Center, Shahid Beheshti University of Medical Sciences, Tehran, Iran*; 2 *Department of Mycobacteriology and Pulmonary Research, Pasteur Institute of Iran, Tehran, Iran*; 3 *General Practitioner (GP), Ebne-sina Medical Center (EMC), Tehran, Iran*; 4 *Medical Students Research Committee, Shahed University, Tehran, Iran*

**Keywords:** Permcath, Endocarditis, Hemodialysis, COVID-19, Acute renal failure

## Abstract

**Background & Objective:**

COVID-19 infection is known to affect the kidneys, potentially resulting in Acute Kidney Injury (AKI). In some patients, however, renal failure may necessitate hemodialysis following a COVID-19 infection. Pneumothorax, hemothorax, cardiac arrhythmias, and endocarditis are some complications associated with Permcath insertion, a type of vascular access used in hemodialysis.

**Case Presentation:**

In this report, we present a 29-year-old man who suffered from endocarditis following hemodialysis treatments through a Permcath because of acute renal failure following COVID-19. Removing the large masses (3×3 cm) inside the right atrium (RA) and taking out the Permcath was completed surgically.

**Conclusion:**

A suitable Permcath and its accurate insertion are recommended to provide prompt AKI treatment and prevent further complications, such as endocarditis and thrombosis.

## Introduction

A Permcath is a flexible tunneled tube that requires a medical procedure for placement within a blood vessel, typically in the neck region. It is primarily used as a long-term alternative and enduring for vascular access in individuals with End-Stage Renal Disease (ESRD) who require hemodialysis. Some complications associated with this medical device include pneumothorax, hemothorax, arrhythmias, and endocarditis (1). The Coronavirus Disease 2019 (COVID-19) affects the kidneys, resulting in a well-known complication known as Acute Kidney Injury (AKI), which generally resolves in most cases. AKI is characterized by an increase in Creatinine (Cr) of equal to or greater than 50% from its baseline level and/or a reduction in the Glomerular Filtration Rate (GFR) of equal to or greater than 25% and/or a decrease in urine output to less than 0.5 ml/kg/h for a duration of six hours or longer (2). The evidence indicates potential direct invasion of the kidney by SARS-CoV-2, leading to AKI in COVID-19 patients (3). Patients experiencing Persistent Severe AKI (PS-AKI), defined as AKI lasting for at least three days, are linked to poorer prognoses and higher mortality rates compared to COVID-19 patients without AKI or with nonpersistent AKI (4). The evidence regarding the development of Focal Segmental Glomerulosclerosis (FSGS) after recovery from COVID-19 is limited; however, it has been reported that approximately 13% of patients who experience FSGS after COVID-19 infection eventually become dialysis-dependent (5). Additionally, only a few patients developed endocarditis due to Permcath insertion, with subsequent recovery. However, to the best of our knowledge, there have been no reported cases of endocarditis due to Permcath insertion in patients with PS-AKI dependent on hemodialysis following COVID-19 with FSGS identified in renal biopsy (6). This case report describes a 29-year-old male patient who developed endocarditis following several hemodialysis treatments through Permcath due to COVID-19-induced renal failure.

## Case Presentation

A 29-year-old man with a known case of ESRD was admitted to the Emergency Department (ED) after two days of fever and worsening dyspnea. He was a 6-pack/year smoker with no record of underlying disease. Renal failure was detected 1–2 weeks after his diagnosis of COVID-19 infection, which occurred six months before his ED admission. At that time, a renal biopsy was performed, which revealed FSGS, a pathological lesion associated with post-COVID renal injury. Hemodialysis was performed via a vascular access catheter two times a week, with each session lasting four hours. The vascular catheter used was a Permcath, which was inserted into the right subclavian vein and finally through the Right Atrium (RA). The insertion was performed by an experienced vascular surgeon according to the aseptic technique four months ago. Although the patient was informed about the possible risks associated with Permcath, such as infection, he declined the timely creation of an arteriovenous fistula for personal reasons. His drug history included furosemide, carvedilol, atorvastatin, prednisolone, and cyclosporine.

At the time of ED presentation, the patient had stable vital signs (Blood Pressure (BP): 109/73 mmHg; heart rate: 79 beats/minute; respiratory rate: 20 respirations/minute; oxygen saturation: 93% on room air; temperature: 38.2 °C, and Glasgow Coma Scale: 15/15). Upon physical examination, generalized edema, including ascites, and preorbital and lower limb edema were evident. Additionally, heart and lung auscultation revealed a systolic murmur (III/VI) and crackles in the right lower lung, respectively. The initial laboratory findings showed White Blood Cell count (WBC): 12000 cells/mm^3 ^(4000–10000 cells/mm^3^), Hemoglobin (Hb): 9.3 g/dl (13.2–17.5 g/dl), platelet: 131000 cells/mm^3^ (150000–450000 cells/mm^3^), sodium: 139 meq/L (135–148 meq/L), potassium: 3 meq/L (3.5–5.5 meq/L), Cr: 2.24 mg/dl (0.7–1.4 mg/dl), urea: 47.9 mg/dl (15–44 mg/dl), uric acid: 2.4 mg/dl (3.6-7.2 mg/dL), erythrocyte sedimentation rate: 60 mm/hr (up to 15 mm/h), C-reactive protein: 5 mg/L (up to 6 mg/L), Albumin (Alb): 2.2 g/dl (3.5 to 5.0 g/dL), bilirubin total: 0.55 mg/dl (0.2-1.2 mg/dl), bilirubin direct: 0.12 mg/dl (up to 0.25 mg/dl), Prothrombin Time (PT): 12.9 sec (12.5–14.5 seconds), International Normalized Ratio (INR): 1.11 (1.00–1.10), Partial Thromboplastin Time (PTT): 25 sec (25-35 seconds), aspartate aminotransferase (AST): 17 U/L (up to 40 U/L), Alanine Aminotransferase (ALT): 13 U/L (up to 45 U/L), Alkaline Phosphatase (Alk P): 143 U/L (80-306 U/L), Hepatitis B Surface Antigen (HBS Ag): negative, Hepatitis C Virus Antibody (HCV Ab): nonreactive, Human Immunodeficiency Virus Antibody (HIV Ab): nonreactive, troponin: 0.02 ng/ml (up to 0.06 ng/ml), Creatine Phosphokinase (CPK): 55 IU/L (up to 195 IU/L), Creatine Kinase Myocardial Band (CK-MB) mass: 2.1 ng/ml (up to 4 ng/ml), Lactate Dehydrogenase (LDH) serum: 803 U/L (230-480 U/L), blood sugar: 88 mg/dl (up to 140 mg/dl), total cholesterol: 232 mg/dl (< 200 mg/dL), triglyceride: 421 mg/dl (< 150 mg/dl), Severe Acute Respiratory Syndrome Coronavirus 2 Immunoglobulin M (SARS-COV_2 IgM): 0.1 AU/ml (negative< 0.9), Severe Acute Respiratory Syndrome Coronavirus 2 Immunoglobulin G (SARS-COV_2 IgG): 2.3 AU/ml (negative< 0.9), urine protein: +3 (normal= negative), urine culture: no growth. The sample was taken for blood culture three times at one-hour intervals before administering antibiotics. Also, the result of the COVID-19 PCR test was negative after admission.

**Figure 1:**
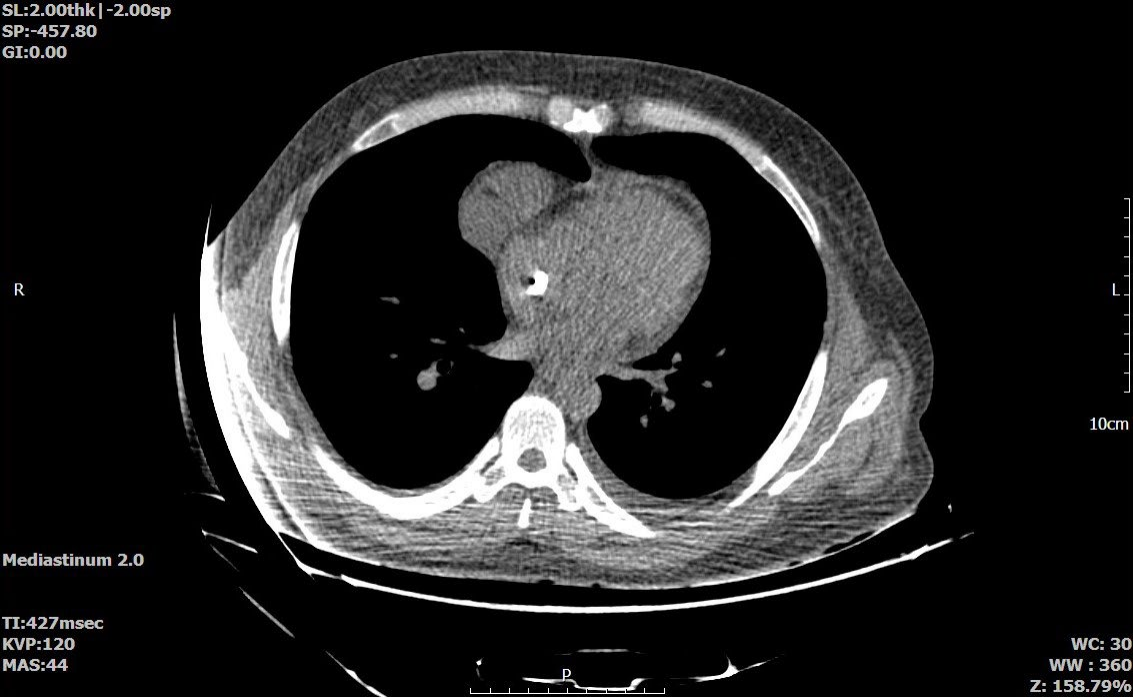
Incidentally found Morgagni hernia with the tip of the Permcath in the right atrium

In further evaluations, echocardiography revealed a large fixed mass with a mobile pedicle in the RA-free wall and another large fixed mass around the catheter in the Superior Vena Cava (SVC). Additionally, vegetation in the tricuspid valve was observed, which was subsequently confirmed via Transesophageal Echocardiography (TEE). His ejection fraction was 60%, and other indices were within the normal range. Additionally, a chest Computed Tomography (CT) scan revealed mild bilateral pleural and pericardial effusion with a Permcath tip evident in the RA. Also, diffuse centrilobular ground glass patterns in the upper lobes were evidence of long COVID-19 pulmonary injury. Additionally, a Morgagni hernia on the right side was found, as shown in Figure 1. For further evaluation of the lungs regarding long COVID-19 injury, spirometry was performed, which revealed an FEV1:68% FVC:57% FEV1/FVC:94%, which was indicative of a moderate to severe restrictive pattern. After careful evaluation of the patient's medical history and test results, other causes of acute kidney injury (AKI), such as prerenal or postrenal factors, were excluded. Given the recent history of COVID-19 infection, intrinsic AKI due to viral infection was considered a possible etiology.

The initial management included empirical antibiotic therapy with meropenem (1 g every 8 hours), vancomycin (1 g every 12 hours), and ciprofloxacin (400 mg every 12 hours). In addition to his previously used medications, including prednisolone and cyclosporine, a heparin drip with a dose of 1200 units per hour was administered. Additionally, the hemodialysis was increased to three times per week, and the patient was closely monitored. After two days, the blood culture result was reported to be positive for *Pseudomonas aeruginosa,* and based on the antibiogram, it was sensitive to meropenem and ciprofloxacin. Therefore, intravenous antibiotic treatment was continued without vancomycin.

**Figure 2:**
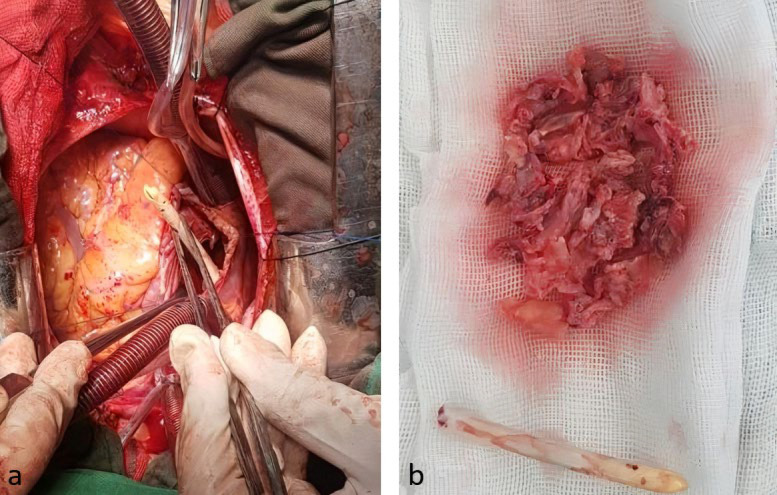
Permcath and clots: (a), Tip of the Permcath in the right atrium during heart surgery. (b), Large masses of clots were removed after surgery.

Due to the risk of migrating blood clots and vegetation to the lungs and causing pulmonary embolism, surgical treatment was chosen. For surgical treatment, the operation of removing the masses was performed by sternotomy and beating heart bypass surgery. The wall of the RA was opened, where two large masses were found. One (3×3 cm) was attached to the interatrial septum and another (3×2.5 cm) was attached to the RA-free wall, both of which were removed. Additionally, another mass that was attached to the Permcath (and inside the SVC) and another clot on the tricuspid valve were also removed. Figure 2 shows the Permcath and clots during and after surgery. The vegetation and the Permcath tip were sent for microbiological culture and cytopathological examination. Pathology revealed thrombosis and blood clots were reported a positive culture of *Pseudomonas aeruginosa *was reported. Moreover, the pericardial fluid was sent for biochemical and cytological analysis, which revealed the following characteristics: WBC: 300, Red Blood Cell (RBC): 500, Polymorphonuclear (PMN) cell: 88%, Mononuclear (MN) cell: 12%, fluid glucose: 388 mg/dL (normal pericardial fluid glucose is usually close to serum levels of 60–100 mg/dL), smear: WBC= 3-4, Alb: 2 g/dl, and fluid culture: no growth. During surgery, the Central Venous (CV) line was inserted into the left jugular vein to provide access for dialysis.

After surgery, TEE was repeated, which revealed no abnormalities. Additionally, during the follow-up echocardiography five days after surgery, no residual mass in the RA was observed, and the Left Ventricular (LV) ejection fraction was 55%. Despite having no further complications, however, he continued to receive hemodialysis due to renal failure using an inserted CV line. After 8 days, the patient was discharged with the following laboratory values: Hb: 10.8, INR: 2.56, PT: 30, PTT: 37, and Cr: 1.22. Antibiotic therapy was continued with oral ciprofloxacin (500 mg every 12 hours) for the next four weeks. Warfarin was prescribed three days after surgery instead of heparin, which was discontinued, and the INR target of 2.5-3 remained during the follow-up. Additionally, his previous drugs, such as prednisolone, cyclosporine, carvedilol, and atorvastatin, were continued. During the three-month follow-up, his echocardiography and renal function laboratory tests were stable.

## Discussion

From a pathological perspective, this case demonstrates how catheter-related trauma and infection can result in intracardiac thrombosis and vegetations, which were confirmed through cytopathological evaluation and culture. Furthermore, the renal biopsy findings of FSGS establish a pathological basis for the patient’s dialysis dependence after COVID-19. The integration of histopathology (renal biopsy), cytopathology (clot and catheter tip analysis), and biochemical fluid assessment was essential in confirming the underlying mechanisms of organ damage. This highlights the importance of pathology in guiding both diagnosis and treatment decisions in complex post-COVID hemodialysis patients. Endocarditis and atrial thrombosis are serious and potentially life-threatening complications associated with Permcath insertion in hemodialysis patients. In this context, the most common causative organism for endocarditis is *Staphylococcus aureus*, accounting for a significant proportion of *S. aureus* bacteremias. However, other microorganisms, including *coagulase-negative staphylococci*, *enterococci*, and *viridans group streptococci*, can also contribute to Permcath-related endocarditis (7). In the context of a COVID-19 infection, the possible occurrence of endocarditis may be explained by the level of inflammation triggered by the COVID-19 virus. Moreover, it has been shown that damages to the endocardium induced by inflammation serve as a primary site for the attachment and colonization of pathogenic agents (8). Similarly, the excessive inflammatory reaction triggered by COVID-19 has been extensively characterized by cytokine storm pathways; thus, it might result in endocardial damage of the heart valves (9). Early identification allows timely initiation of appropriate treatment, including intravenous antibiotic therapy. Despite advancements in treatment strategies, endocarditis related to Permcaths still has high mortality rates ranging from 30% to 50%, making complete recovery challenging (10). Therefore, healthcare providers must remain vigilant and adopt evidence-based prophylactic and management strategies to improve patient outcomes. Although our patient experienced prominent improvement after treatment for endocarditis, renal failure which required regular hemodialysis, negatively impacted his quality of life. According to Sousa *et. al.*'s study, a catheter placed in the right jugular vein resulted in a bloodstream infection caused by *Candida albicans*. The vegetation, which was probably related to the catheter, was removed by surgery for endocarditis treatment (11). In the Gülmez and Aydın study, two individuals with ESRD underwent hemodialysis through a tunneled catheter; one was 56 years old, and the other was 88 years old. Both patients tested positive for *Staphylococcus aureus* bacteremia in their blood samples. Further examination via echocardiography revealed a perivalvular abscess in the elderly patient and freely moving vegetation on the heart valve in the younger patient. Despite plans for surgical intervention, the elderly patient refused surgery and died from refractory shock. Additionally, the second patient died due to refractory shock and LV failure after surgery (12). Additionally, Rezaei Bookani *et. al.* described a patient with ESRD who underwent hemodialysis with a femoral tunneled catheter. This patient developed endocarditis caused by *Corynebacterium jeikeium* (13). Furthermore, Liang and Landry's study explains infective endocarditis caused by *Staphylococcus aureus* due to a Permcath in a 48-year-old male with ESRD (14). Similarly, the research conducted by Chambi-Torres *et. al.* reported a case of *Staphylococcus aureus* endocarditis due to a Permcath in the internal jugular vein of a 60-year-old woman suffering from ESRD and heart failure (15).

Continuous irritation of the right atrium, particularly when the catheter tip impinges upon the IVC–RA junction, is likely responsible for atrial thrombosis. The deep placement of the catheter tip within the right atrium leads to trauma during cardiac contractions, causing jet injury from infusions. This irritation triggers the coagulation cascade and platelet aggregation, contributing to the development of thrombosis (16). Studies have demonstrated a higher incidence of atrial thrombosis when the catheter tip is placed in the right atrium; therefore, it is recommended that the catheter tip be positioned optimally at the junction between the SVC and the RA to minimize the risk of thrombosis (17). Hypercoagulability in hemodialysis patients is another contributor to thrombosis pathogenesis. In this case, the size of the thrombosis was among the largest to have been reported in the literature. In our patient, several mechanisms likely contributed to the formation of large intracardiac masses and endocarditis. The use of immunosuppressive therapy (prednisolone and cyclosporine) may have blunted the host immune response, reducing the ability to clear infection and predisposing to vegetation growth. In addition, COVID-19-associated hypercoagulability and endothelial dysfunction have been well documented and may have amplified the thrombotic potential of the indwelling catheter tip. Continuous mechanical trauma from the catheter tip against the right atrial wall further created a nidus for thrombus deposition and microbial colonization. From a clinical practice perspective, these mechanisms underscore the importance of strict catheter management protocols, including optimal catheter tip placement at the SVC–RA junction, rigorous aseptic technique, and timely transition to arteriovenous fistula whenever possible. Moreover, early echocardiographic evaluation should be considered in post-COVID renal failure patients with suspected infection or unexplained symptoms to allow earlier detection of vegetations or thrombi. At the policy level, this case also highlights the need for clear guideline-based strategies addressing anticoagulation use in hypercoagulable patients, careful balancing of immunosuppressive therapy when infection risk is high, and standardized surveillance protocols to reduce catheter-related complications in vulnerable populations.

AKI was frequently observed amongst COVID-19 patients; however, most patients had a good prognosis with no further renal complications (6). Nevertheless, some patients experienced renal failure after COVID-19 infection and required hemodialysis. Therefore, both the reason and the complication of Permcath insertion were not prevalent. Despite the control of COVID-19 mortality, its complications still have a significant effect on patients. An interesting feature in this case was the near-normal CRP level (5 mg/L) despite clear evidence of bacteremia and intracardiac vegetation. This paradox may be explained by the patient’s ongoing immunosuppressive therapy with prednisolone and cyclosporine, which can blunt the host inflammatory response and thereby suppress CRP elevation. In addition, the timing of CRP measurement relative to antibiotic administration could have influenced the result. This highlights the limitation of relying solely on CRP in immunosuppressed or partially treated patients and underlines the importance of integrating laboratory, imaging, and microbiological data for accurate diagnosis. The pericardial fluid analysis in this case showed an unexpectedly high glucose level of 388 mg/dL. This was clarified as a true measurement in mg/dL, obtained from a sample collected intraoperatively and promptly analyzed. Given that normal pericardial glucose parallels serum levels, and that bacterial or tuberculous pericarditis typically reduces glucose values, such a high value was most plausibly due to contamination with systemic blood glucose in the setting of perioperative hyperglycemia. Taken together with the low WBC count and negative cultures, the profile was more consistent with a transudative effusion rather than an inflammatory or infectious process.

## Conclusion

This case underscores not only the clinical but also the pathological consequences of prolonged Permcath use in post-COVID renal failure. Renal biopsy findings, cytopathological confirmation of vegetations, and pericardial fluid analysis collectively emphasize the role of pathology in understanding and managing such complications. The order of events that occurred in this young adult demonstrates how COVID-19 negatively impacts the quality of life, increases the number of invasive procedures with serious complications, and enhances economic burdens by increasing the costs of hospitalization and treatment. Thus, a comprehensive approach is essential to improve patient outcomes and reduce the incidence of both endocarditis and thrombosis in Permcath patients. We recommend the use of suitable Permcaths with optimal tip placement, regular monitoring of patients, and maintenance of Permcaths to detect infections promptly.

## List of abbreviations

AKI Acute Kidney Injury

Alb Albumin

ALT Alanine Aminotransferase

Alk P Alkaline Phosphatase

AST Aspartate Aminotransferase

BP Blood Pressure

CK-MB Creatine Kinase Myocardial Band

COVID-19 Coronavirus Disease 2019

Cr Creatinine

CPK Creatine Phosphokinase

CT Computed Tomography

CV Central Venous

ED Emergency Department

ESRD End-Stage Renal Disease

FEV1 Forced Expiratory Volume in 1 Second

FSGS Focal Segmental Glomerulosclerosis

FVC Forced Vital Capacity

GFR Glomerular Filtration Rate

Hb Hemoglobin

HBS Ag epatitis B Surface Antigen

HCV Ab Hepatitis C Virus Antibody

HIV Ab Human Immunodeficiency Virus Antibody

IgG Immunoglobulin G

IgM Immunoglobulin M

INR International Normalized Ratio

LDH Lactate Dehydrogenase

LV Left Ventricle

MN Mononuclear

PCR Polymerase Chain Reaction

PMN Polymorphonuclear

PS-AKI Persistent Severe Acute Kidney Injury

PT Prothrombin Time

PTT Partial Thromboplastin Time

RA Right Atrium

RBC Red Blood Cell

SVC Superior Vena Cava

TEE Transesophageal Echocardiography

WBC White Blood Cell
